# UNRAVELING COMORBIDITY PREVALENCE AND PM2.5 EXPOSURE AMONG MIDDLE-AGED AND ELDERLY: A SPATIAL-TEMPORAL ANALYSIS

**DOI:** 10.1093/geroni/igad104.3795

**Published:** 2023-12-21

**Authors:** Linjiang Wei, Liangwen Zhang, Ya Fang

**Affiliations:** Xiamen University, Xiamen, Fujian, China (People’s Republic); Xiamen University, Xiamen, Fujian, China (People’s Republic); Xiamen University, Xiamen, Fujian, China (People’s Republic)

## Abstract

**Objective:**

This study describes regional differences and dynamic changes in the prevalence of comorbidities among middle-aged and elderly people with chronic diseases (PCMC) in China from 2011-2018, and explores distribution patterns and the relationship between PM2.5 and PCMC, aiming to provide data support for regional prevention and control measures for chronic disease comorbidities in China.

**Methods:**

This study utilized CHARLS follow-up data for ≥45-year-old individuals from 2011, 2013, 2015, and 2018 as research subjects. Missing values were filled using the random forest machine learning method. PCMC spatial clustering investigated using spatial autocorrelation methods. The relationship between macro factors and PCMC was examined using Geographically and Temporally Weighted Regression, Ordinary Linear Regression, and Geographically Weighted Regression.

**Results:**

PCMC in China showing a decreasing trend. Hotspots of PCMC appeared mainly in western and northern provinces, while cold spots were in southeastern coastal provinces. PM2.5 content was a risk factor for PCMC, the range of influence expanded from the southeastern coastal areas to inland areas, and the magnitude of influence decreased from the southeastern coastal areas to inland areas.

**Conclusion:**

PM2.5 content, as a risk factor, should be given special attention, taking into account regional factors. In the future, policy-makers should develop stricter air pollution control policies based on different regional economic, demographic, and geographic factors, while promoting public education, increasing public transportation, and urban green coverage.

